# Text Book of Anatomy for Students

**Published:** 1845-04

**Authors:** 


### Text Book of Anatomy for Students. By *Alexander Jardine Lizars*, M.D. Professor of Anatomy in the Marischal College and University of Aberdeen. Parts 2 and 3. 1844.

Under the unpretending title of a Text Book, Dr. Lizars has rendered a very acceptable service, by giving to the student a clear exposition, not only of the descriptive anatomy of the body, but likewise a concise enumeration of the more important points connected with the minute structure of the several organs and their actions. We have always thought it desirable that, even in elementary works, some reference should be made to structural anatomy and physiology; not only because the tedium which attaches to a bare description of the processes of a bone or the attachments of a muscle, is considerably relieved by knowing something of that beautiful mechanism which their intimate texture reveals, but more especially because the future practitioner will, in the investigation of disease, find more demand made upon his knowledge of what has been well-called *physiological anatomy*, than upon his acquaintance with the topographical and surgical anatomy, all-important as they are in themselves.

In the work before us, we are also glad to perceive that the author has incorporated the more essential part of the various discoveries and additions by which the science of anatomy has of late years been enriched; so that, after going through a minute account of the divisions of an artery or the twigs of a nerve, the reader is refreshed by arriving at some novel and interesting sketch from which he cannot fail to derive pleasure and instruction. But in thus rendering available the results of modern discovery, the plan of the work is never sacrificed.

The objects of the author are thus stated :—

" Having had ample opportunities of observing how much Students are generally occupied during lecture time in taking notes of the Anatomical Demonstrations which are then being delivered, I have prepared the present Treatise with the view of saving them that trouble."

" This little work will enable the student to follow lectures on Anatomy with, I trust, every possible facility; and will supply him, at the termination of his studies, when his time is invaluable, with the readiest means of reviewing this branch of Science within the narrowest limits. In the execution of my task I have steadily aimed at the greatest degree of conciseness compatible with the requisite minuteness, and with accuracy; and utility has been the great and constant object of my ambition."—*Preface.*

Although the general character of the work before us, enables us to express a favourable opinion of its merits, yet there are some occasional errors and omissions which require correction. Among the former, we regard the statement that the rete mucosum "is unorganised, and is evidently a secretion of the dermis." (p. 275.) There can be no doubt, on the contrary, that the *rete Malpighii* is composed of distinct cells, which are doubtless extra-vascular; but to call such a structure in the present day unorganised, or a secretion, is incorrect. Again, the structure of the cornea is thus given—" it is composed of six lamellæ, having a fluid in minute quantity between them." (p. 291.) Now, although a minute account of texture would have been misplaced in a text-book, yet, in an enumeration of the component parts of an organ, all that do exist should have been named; but we have here no notice of the epithelium covering the outer surface, nor of the membrane of Demours and layer of tessellate epithelium which exist upon the posterior surface. These, and some other omissions of a similar kind are, however, of a trifling nature, and are redeemed by concise and yet comprehensive descriptions of the several organs.

The anatomy of the brain is illustrated by some tabular classifications, which may assist the student in acquiring a technical acquaintance with the names and relations of the fissures, lobes, &c.

The structure of the spinal cord is well given, and many of the more recent views respecting its minute texture and functions are briefly expressed. The physiology of this important organ is thus described :—

" The Functions of the spinal cord are, 1, Nervous impression,—2, Nervous power,—3, Sensation,—4, Volition.

"1. *Nervous Impression.* Impression made upon the various parts of the trunk and limbs may be transmitted to the cord, without the consciousness of the individual; as happens in diseases where the mental faculties are suspended.

"2. *Nervous Power,* or nervous energy, is the function whereby a stimulus is sent to certain muscles so as to excite their contractions. This either follows the preceding, or volition. The movements of the various muscles connected with the organs of relation are produced by this power; and those also it is said of the muscular fibres of the organs of nutrition. That it exists independent of volition is proved by movements being produced against the will; also by certain diseases of the cord, wherein the individual has the *will,* but not the *power* to effect the movement; and by those affections of the brain, where volition is lost, while this function remains.

"3. *Sensation* is the perception of an impression of which the individual is conscious. The cord assists in the performance of this function only so long as its connection with the brain is maintained.

"4. *Volition,* the function which at certain times precedes the actions of the voluntary muscles, is performed by the cord only so long as it is connected with the brain." P. 352.

The third and concluding part of the work contains the organs of nutrition and generation, and the style in which these subjects are treated will be comprehended by a few selections.

The teeth are, by Dr. Lizars, very properly described with the digestive organs, with which they are not only connected in function, as instruments for masticating the food, but by their relation to the mucous membrane. The dental organs, if examined only in the adult and in the human body, would probably be regarded, as indeed they often have been, as appendages to the skeleton; but comparative and developmental anatomy teach us, what Hunter had long since inferred, that they are parts of the tegumentary membranes. Thus in many animals they are unconnected with the jaws, being placed in numerous fishes on the tongue or in the pharynx; and in several molluscous and articulate animals they are even implanted on the mucous surface of the stomach, of which a familiar example is furnished by the common lobster. Again, the valuable researches of several late observers, especially those of Mr. Goodsir, have shown that the teeth are formed, not like the bones, but from papillæ, like the hairs implanted in the skin.

With the stomach and intestinal canal, in addition to the usual descriptive anatomy, a brief account is given of the epithelium, villi, and the various glands, such as the gastric, duodenal, those of Peyer, Lieberkühn, and so forth. There is also appended a sketch of the physiology of each organ. The organization of the liver is well described, according to the views of Mr. Kiernan, which, although rendered doubtful in some parts by the recent inquiries of Henle, Bowman, Weber, and Krukenberg, are still deserving of close attention.

In the section upon the kidney there is an omission which the author would do well to rectify in another edition; we allude to the absence of all notice of the important researches of Mr. Bowman, which have thrown an entirely new light upon the character of the Malpighian bodies, and especially upon the renal circulation. The views of Müller and other German physiologists, who attribute free cœcal extremities to the cortical tubules, are also adopted, although the subsequent researches of Professor Owen and others have rendered it very probable that such a disposition does not really exist.

The Chapter on the organs of respiration contains a recapitulation of the several structures of the lungs. The part relating to the bronchial tubes is at once concise, clear, and correct, although we differ from Dr. Lizars when he says, adopting the views of Reisseissen, " each minute tube terminates in a vesicle or cul-de-sac very slightly if at all dilated, and a number of these are collected together, so as to form what is termed a bronchial lobulus." (p. 528). We refer our readers for what we believe to be a more correct statement of this interesting part of structural anatomy to our notice of Dr. Carpenter's treatise on Physiology.

A full account is given of the distribution of the vascular system, but as this admits of little novelty, one or two extracts will be sufficient to show the plan the author has adopted of simplifying the study of these parts of the body. The distinctive character of the capillaries are well-defined in the following passage;—" they (the small arteries) ultimately terminate in a series of minute vessels named *capillaries*, the walls of which are of extreme tenuity. The size of the different capillaries varies from $\frac{1}{1000}$ to $\frac{1}{5000}$ of an inch; but each capillary is of the same calibre throughout its whole length; they neither diminish, like arteries, nor increase, like veins, and they frequently unite or anastomose with each other, so as to form a net work, by which the distribution of the blood is facilitated." (p. 535).

A tabular summary is appended to the account of all the blood-vessels, nerves, &c. which is well calculated to facilitate the progress of the student.

A description of the cavities, textures, and action of the heart, arteries and veins, is prefixed to the account of the blood-vessels. The minute anatomy of the heart is full and comprehensive, but we think that the author should, instead of tracing the carneous fibres from the interior of the organ, have followed them in the mode which is not only the most obvious but also the only effective one, namely, from the exterior, inwards.

The ganglionic or sympathetic system of nerves is considered after the organs of circulation, and we have the following exposition of the more essential parts of this system, which, for correct and judicious views, might compete with many more elaborate and pretending productions.

" STRUCTURE OF THE GANGLIONIC NERVES. These nerves are composed of three different kinds of threads or fibres, to which the names of sensific, motific, and organic are applied.

" *The Sensific Fibres* are obtained from the cerebro-spinal axis, through the medium of its nerves, a few being furnished by some of the cerebral, and the majority by the spinal nerves, the posterior roots of all of the latter giving off fibres. These are of a white colour and tubular form, the nervous matter being contained in minute canals formed of fine cellular tissue.

" *The Motific Fibres* are also derived from the cerebro-spinal axis, certain of the cerebral nerves giving a few, and the anterior roots of all the spinal nerves the greater number. In form and colour they resemble the last.

" *The Organic Fibres* arise in the ganglia of this system. They are much more minute than the two preceding fibres, and, when examined with the microscope, are found to differ from them, in not being composed of a tube with nervous matter in the interior, but in being of a homogeneous structure. They are also pale, transparent, and of a slightly reddish tint, and have their surface studded, here and there, with small bodies of a round or oval form."

"Structure of the Ganglia. They are of a reddish-gray colour, and composed of two coats or tunics, in the interior of which are found globules, nervous fibres, and blood-vessels.

"*The Tunics* are distinguished into an external, composed of condensed cellular tissue, and an internal, more delicate than the preceding, which sends off processes from its inner surface into the substance of the ganglion.

"*The Globules* are small bodies, nearly spherical in form, and having caudal or tail-like processes. Delicate fibres extend between them and connect them. The greater number of anatomists consider them identical with the cineritious substance of the cerebro-spinal axis.

"*The Nervous Fibres* found in the ganglia may be arranged into the sensific, motific, and organic. The arrangement of these in the ganglia is most intricate, and is consequently made out with much difficulty. The sensific and motific fibres, which have been already mentioned to be derived from the cerebro-spinal axis, and which, in the ganglionic nerves, run parallel to, and are in apposition with one another, in the ganglia separate from each other, repeatedly unite and again separate, and thus form a most complicated net-work or plexus, the intervals of which are occupied chiefly by the globules. With the exception, then, of their separation, the sensific and motific fibres undergo no change in the ganglia; they are not increased in size in passing through them, and they have no further connexion with the globules than merely running between them. The organic fibres, on the other hand, are said to commence in the ganglia, in which they arise from the caudal processes of the globules; when such fibres then traverse the ganglia, they are frequently increased in size by the addition of new fibres derived from the globules." P. 804.

We regret that our space does not enable us to insert the excellent remarks of Dr. Lizars on "the functions of the ganglionic system," they are, however, well worthy of perusal.

The work is brought to a conclusion by a description of the reproductive organs, in which the latest researches are introduced. Those of our readers who are acquainted with the writings of Baer, Purkinje, W. Jones, R. Wagner, Barry, and others, will recognise the accuracy of the subjoined account of the mammiferous ovarium ovum.

"The Ova, ovula, or vesicles of Baer, by all of which names they are known, are the germs from which the embryo or offspring are produced. It has been said that they are suspended in the Graafian vesicles by retinacula; in the smaller vesicles they are placed nearly in the centre, but in those which are fully formed, they are situated close to the inner surface or epithelium. Each is of a spherical shape, is about $\frac{1}{10}$th of a line, and presents the following parts: First, the exterior is covered with a dense layer of granules, which form what is termed the *tunica granulosa;* these granules are similar to those contained in the fluid of the Graafian vesicle, and those which enter into the formation of the membrana granulosa of that body, and the retinacula. Secondly, within the tunica granulosa is another thick tunic, named the *zona pellucida*; it is composed of an albuminous mass, enclosed in a membrane of extreme tenuity; it is transparent, and when seen under the microscope exhibits the appearance of a bright ring. Thirdly, contained in the zona pellucida is a mass termed the *yolk*, consisting of granules or cells and fat globules. Fourthly, lodged at first in the middle, but afterwards on one side of the yolk, is a minute body, to which the name of *germinal vesicle*, or germinal vesicle of Purkinje has been given; its diameter is about $\frac{1}{60}$th of a line; and its contents are transparent, except at one point. Fifthly, and lastly, within the germinal vesicle and close to its surface, is an opaque spot, whose diameter is only about $\frac{1}{700}$ or $\frac{1}{300}$ of a line: this is termed the *germinal spot,* or germinal spot of Wagner. The germinal vesicle and spot form the essential parts of the ovum; it is at the latter that the development of the embryo commences. These parts of the ovum will be impressed upon the memory by arranging them in a tabular form." P. 895.

The extracts we have given will enable our readers to judge of the general scope and execution of the "Text-Book," which we can with much satisfaction recommend to those for whose assistance it is more especially designed, feeling assured that the student will here find, in addition to the ordinary contents of works of this class, a large number of important facts relating to the higher branches of anatomical science.

